# Progress in the diagnosis and treatment of post-traumatic hydrocephalus

**DOI:** 10.3389/fneur.2026.1821964

**Published:** 2026-07-15

**Authors:** Tao Yang, Song Zhang, Linguo Bai, Yijing Xie, Shanshan Liu

**Affiliations:** 1 Department of Neurosurgery, The Second Affiliated Hospital of Guizhou University of Chinese Medicine, Guiyang, Guizhou, China; 2 Department of Pathology, Guizhou Hospital of Guangdong Provincial Hospital of Traditional Chinese Medicine, Guiyang, Guizhou, China

**Keywords:** neuroinflammation, pathophysiologic mechanisms, risk factors, traumatic hydrocephalus, treatment progress

## Abstract

**Objective:**

Post-traumatic hydrocephalus (PTH) is a frequent secondary complication after traumatic brain injury and significantly affects neurological recovery and prognosis. This review summarizes epidemiology, mechanisms, risk factors, diagnostic advances, and treatment strategies to improve clinical recognition and management.

**Methods:**

A narrative literature review was conducted using major medical databases to identify clinical, imaging, and experimental studies addressing incidence, pathophysiology, diagnosis, and therapy of PTH. Relevant findings were synthesized qualitatively.

**Results:**

PTH occurs across injury severities and is associated with subarachnoid hemorrhage, decompressive craniectomy, infection, and advanced age. Proposed mechanisms include cerebrospinal fluid circulation disturbance, impaired absorption, neuroinflammation, and altered intracranial compliance. Diagnostic accuracy has improved with advanced neuroimaging and dynamic cerebrospinal fluid assessment. Therapeutic approaches have evolved from conventional shunting to individualized strategies incorporating endoscopic techniques and adjustable shunt systems.

**Discussion:**

PTH remains underdiagnosed due to heterogeneous presentations. Future efforts should focus on standardized diagnostic criteria and early risk stratification, integration of glymphatic and aquaporin-4 (AQP4) pathways, biomarker- and AI-guided diagnostics, and mechanism-targeted preventive strategies across the acute, subacute, and chronic phases of PTH.

## 1 Introduction

Post-traumatic hydrocephalus (PTH) is a prevalent secondary pathological alteration following traumatic brain injury (TBI), primarily characterized by abnormal accumulation of cerebrospinal fluid (CSF) within the ventricular system, leading to elevated intracranial pressure and neurological deficits (1, 2). The pathogenesis of PTH is complex and may involve an imbalance in CSF secretion and absorption, dysfunction of arachnoid granules, or mechanical obstruction of the ventricular system, particularly in patients with TBI who have experienced decompression of the desmoid valve or concurrent intracranial hemorrhage (3, 4). Epidemiological studies indicate that the prevalence of PTH varies widely in the TBI population (0.7%−51.4%), with risk factors including advanced age, severe craniocerebral injury, and CSF leakage (5, 6).

The diagnosis of PTH depends on imaging techniques (e.g., CT or MRI) with clinical manifestations, including gradual cognitive decline, gait irregularities, or decrease in consciousness (3, 7). The primary method for the treatment of PTH remains ventriculoperitoneal shunt (VPS), which has a complication risk of up to 30%, including shunt blockage, infection, and overdrainage (8, 9). Recent research has sought to impede the development of CSF by altering CSF dynamics by pharmacological interventions (e.g., statins), although their long-term effectiveness has to be validated (10, 11). In addition, novel smart shunt devices and biomarker-driven personalized diagnostic approaches present promising avenues for PTH therapy; yet, they continue to encounter hurdles related to technological maturity and practical implementation (7, 11, 12).

Despite the progress made in clinical research on PTH, its diagnostic criteria, prognostic assessment, and treatment optimization remain subject to debate. The process of neurological recovery in postoperative shunt-dependent patients remains little understood, and there is an absence of interdisciplinary and integrated care models for secondary brain injuries (5, 13). Based on the latest clinical evidence, this article systematically reviews the definition and epidemiology, pathophysiological mechanisms, diagnostic advances, therapeutic challenges, and future research directions of PTH, to provide a theoretical foundation for improving clinical practice. The scope of this review was deliberately confined to three closely related domains (definition and epidemiology, pathogenesis and risk factors, and treatment) because these areas have undergone the most rapid progress in the past five years and constitute the foundation on which diagnosis, animal modeling, cellular research, and complication management ultimately depend. Topics such as preclinical models and detailed complication management are addressed elsewhere in dedicated reviews and were therefore not duplicated here. To improve mechanistic clarity, the present revision organizes pathogenesis along an acute, subacute, and chronic temporal axis (summarized in Table 1) and explicitly links each mechanism to potential therapeutic and preventive targets.

**Table 1:** A temporal framework linking pathogenesis, clinical features, and candidate therapeutic targets across the acute, subacute, and chronic phases of post-traumatic hydrocephalus (PTH).

Phase	Dominant mechanisms	Clinical and imaging features	Candidate therapeutic targets
Acute (0–7 day)	Blood–brain barrier disruption; intraventricular and subarachnoid hemorrhage; choroid plexus CSF hypersecretion (TLR4/NF-κB, NKCC1)	Raised intracranial pressure, depressed consciousness; CT or MRI showing haematoma, early ventricular distension	Haematoma evacuation and external ventricular drainage; early anti-inflammatory therapy (TLR4/NF-κB, NLRP3); iron chelation (deferoxamine)
Subacute (1–8 week)	Microglial and astrocyte activation; AQP4 depolarisation; arachnoid fibrosis and impaired CSF absorption; post-DC compliance shifts	Progressive ventriculomegaly (Evans index >0.3), subdural effusion, fluctuating consciousness; rising S100B and neurofilament light chain	Early cranioplasty; AQP4 modulation; SGK1 inhibitors; biomarker-guided shunt timing
Chronic (>8 wk)	Glymphatic stagnation; periventricular white-matter injury; possible accumulation of amyloid-β and phosphorylated tau; genetic susceptibility (ciliary genes)	Gait disturbance, cognitive decline, urinary symptoms; DTI changes in periventricular white matter; shunt-dependent ventriculomegaly	VPS or ETV (individualized); glymphatic restoration and sleep optimisation; neuroprotection and rehabilitation; genetic and biomarker-based stratification

PTH, post-traumatic hydrocephalus; TBI, traumatic brain injury; CSF, cerebrospinal fluid; VPS, ventriculoperitoneal shunt; CT, computed tomography; MRI, magnetic resonance imaging; DC, decompressive craniectomy; GCS, glasgow coma score; ISS, injury severity score; ETV, endoscopic third ventriculostomy; VAS, ventriculoatrial shunt; LPS, lumboperitoneal shunt; VVS, ventriculovesical shunt; TGF-β, transforming growth factor-beta; IL-17A, interleukin-17A; IL-1β, interleukin-1 beta, TNF-α, tumor necrosis factor-alpha; IL-18, interleukin-18; OR, odds ratio; AI, artificial intelligence; GOS, glasgow outcome scale.

## 2 Definition and epidemiologic features of PTH

The concept of PTH has evolved through many stages since its clinical characteristics were first described by Dandy in 1914 (14, 15). Initial research, grounded on the idea of CSF circulation, characterized PTH as ventricular enlargement resulting from impaired CSF secretion, absorption, or circulatory routes (14, 15), a mechanical obstruction paradigm that prevailed in the field for decades. Advances in neuroimaging and in-depth investigations of pathological mechanisms have revealed that PTH is not solely associated with traditional mechanisms like arachnoid granule dysfunction, but is closely related to post-traumatic inflammatory responses, modifications in brain tissue compliance, and dysregulation of CSF osmolarity (2, 14, 16, 17). In recent years, several academics have introduced the notion of post-traumatic brain microenvironmental remodeling, highlighting the critical roles of blood-brain barrier breakdown, glial cell activation, and neuroinflammatory cascades in ventricular dilatation (7, 16). Some investigations have distinctly classified progressive ventricular enlargement following debulking decompression as a secondary PTH subtype, since it may include a particular pathophysiological mechanism involving the dynamic equilibrium of cranial volume-pressure (3, 18). Current consensus requires that a diagnosis of PTH meets three core criteria: a definitive history of craniocerebral trauma, imaging-verified progressive ventricular dilatation (Evans index > 0.3), and the exclusion of alternative non-traumatic causes (1, 5, 19), thereby preserving the conventional diagnostic framework while incorporating findings from contemporary molecular pathology research (17, 20).

The prevalence of PTH, a frequent consequence following TBI, exhibits considerable variability based on the research population and variations in diagnostic criteria. The reported incidence of PTH reported in the literature varies significantly, ranging from approximately 0.7%−51.4% among patients with TBI (1, 5). This variation may stem from differing criteria for hydrocephalus assessment and study inclusion across various medical centers (21). Bagherzadeh et al. (22) analyzing 22 studies from PubMed, Scopus, Embase and Web of Science databases (*n* = 2,888) determined a combined incidence of PTH of 20.5%, which represents the overall incidence of PTH. In addition, PTH is very uncommon in child patients with TBI, occurring in just 0.9% of cases (23) and its incidence is reduced by early cranioplasty (24). These data highlight the need to standardize diagnostic criteria and follow-up duration, therefore diminishing research variation and establishing a foundation for healthcare resource allocation and health economic evaluation.

## 3 Pathogenesis and risk factors

### 3.1 Pathogenesis

#### 3.1.1 CSF kinetic imbalance and secretion-absorption disorder

The main pathological mechanism of PTH involves disruption of the dynamic balance of CSF secretion, circulation, and absorption (25, 26). CSF is typically produced by the choroid plexus, circulates through the ventricular system, and is absorbed into the venous system through the arachnoid granulations. Trauma-induced impairment of the blood-brain barrier can result in aberrant osmotic pressure, promoting overproduction of CSF (14, 27). Following craniocerebral trauma, blood-brain barrier disruption, blockage of the ventricular system, and subarachnoid adhesions may result in compromised CSF circulation (14, 28). Research indicates that breakdown of the blood-brain barrier, together with ions released from erythrocyte lysis and hemoglobin derivatives during TBI, might lead to arachnoid granule fibrosis and malfunction, thereby substantially impairing CSF absorption (28, 29).

#### 3.1.2 Secondary pathologic changes

Structural damage and compensatory mechanisms following traumatic brain injury contribute to the advancement of PTH. Trauma directly injures the endothelial cells of the blood-brain barrier, resulting in the extravasation of plasma proteins into the brain parenchyma, which induces vasogenic cerebral edema and elevates the osmotic burden on the CSF (30). The compromise of blood-brain barrier integrity allows inflammatory mediators to enter (e.g., TGF-β, IL-17A) into the ventricular system through the circulation, hence activating microglia and astrocytes, and intensifying neuroinflammation (31). Post-traumatic ventricular separation or ciliary dyskinesia may disrupt CSF laminar flow, resulting in localized vortices and increased hydrostatic pressure, hence exacerbating ventricular enlargement (30, 32). Cranial defects (e.g., after debridement decompression) or intracranial atherosclerosis can reduce brain tissue elasticity and cerebral compliance, resulting in abnormal accumulation of CSF pulse waves within the ventricles and the formation of post-traumatic low-pressure hydrocephalus (33).

#### 3.1.3 Neuroinflammation and immune activation

Neuroinflammation is a key driver in the development of post-traumatic hydrocephalus. Following a brain injury, the degradation of erythrocytes (e.g., hemoglobin) liberates iron ions that activate microglia and choroid plexus macrophages, thereby initiating the Toll-like receptor 4/NF-κB signaling pathway and facilitating the release of pro-inflammatory cytokines (IL-1β, TNF-α)(34, 35), which elicits periventricular inflammatory responses (36), and fosters choroid plexus fibrosis along with augmented CSF secretion (37). The inflammatory cascade directly impairs the tight connections of choroid plexus epithelial cells, resulting in hypersecretion of CSF (27, 38, 39). Clinical studies indicate that NLRP3 inflammasomes are strongly activated in the CSF of patients with post-traumatic hydrocephalus, and their mediated release of IL-18 up-regulates choroid plexus epithelial cell Na^+^/K+/Cl^−^ cotransporter protein activity and increases the rate of CSF production (38, 40). In addition, iron overload-induced oxidative stress exacerbates neuroinflammation and mitochondrial dysfunction by activating the mitochondrial unfolded protein response, thereby creating a positive feedback loop (41). The coagulation cascade and inflammation mutually reinforce one another: post-traumatic coagulation factors (e.g., factor VII, fibronectin) promote microthrombosis and also directly activate microglia and intensify ventricular inflammatory fibrosis (42). Collectively, these mechanisms disturb CSF balance and intensify brain parenchymal damage, resulting in refractory PTH.

#### 3.1.4 Ciliary dysfunction and molecular genetic mechanisms

The synchronized movement of ventricular cell cilia is essential for preserving the directed flow of CSF. Structural abnormalities (e.g., disorganized microtubule arrangement) or dysfunction of the function of ventricular cell cilia following trauma result in CSF stagnation and ventricular dilation (43). Molecularly, RGS22 gene defects disrupt ciliogenesis and polarity establishment by interfering with G protein signaling regulation, inducing hydrocephalus (44). Animal studies demonstrated that mutations in the DNAH5 gene led to the loss of ciliary dynein arms, leading to ciliary dyskinesia and problems of CSF hydrodynamics (45, 46). In addition, downregulation of ULK4 expression impedes ciliogenesis-related pathways (e.g., Hedgehog signaling) and aggravates the advancement of post-traumatic hydrocephalus (46). Clinical investigations indicate that individuals with mutations in ciliopathy-related genes (e.g., CEP290, CCDC39) exhibit a heightened likelihood of developing post-traumatic hydrocephalus, implying an interplay between genetic predisposition and environmental influences (46, 47). Nevertheless, several research studies indicate that ciliary dysfunction (e.g., primary ciliary dyskinesia) has a weaker association with PTH in humans (48). Consequently, ciliary malfunction and genetic processes of hydrocephalus require further investigation for validation.

#### 3.1.5 Glymphatic dysfunction and aquaporin-4-mediated clearance impairment

Beyond classical bulk-flow circulation, the glymphatic system mediates perivascular clearance of solutes from the brain parenchyma and is increasingly recognized as a contributor to chronic post-TBI CSF disequilibrium. Aquaporin-4 (AQP4), polarized to the perivascular astrocytic end-feet, is the principal water channel supporting this clearance pathway. After traumatic brain injury, AQP4 loses its polarized distribution and becomes redistributed across the astrocytic membrane, a phenomenon termed AQP4 depolarisation. This redistribution impairs interstitial fluid drainage, prolongs the residence time of hemoglobin breakdown products and pro-inflammatory cytokines, and sustains periventricular oedema during the subacute and chronic phases of PTH. Persistent glymphatic stagnation may also promote accumulation of phosphorylated tau and amyloid-β oligomers, providing a mechanistic bridge between PTH, post-TBI cognitive decline, aging-related neurodegeneration, and prior cerebrovascular injury. Restoration of AQP4 polarization, modulation of the aquaporin family, and enhancement of glymphatic flow during sleep are therefore emerging as plausible disease-modifying targets in PTH (16).

### 4 Risk factors

There are various risk factors for PTH formation, which can be mainly categorized into primary injury factors, surgical treatment-related factors and other factors.

#### 4.1 Severity of trauma and type of intracranial hemorrhage

The severity of craniocerebral damage is a significant predictor of PTH. Numerous investigations have established that severe subarachnoid hemorrhage, ventricular hemorrhage, hematoma volume, and subdural hematoma substantially raise the risk of PTH (49–52). Traumatic subarachnoid hemorrhage obstructs the CSF circulation system, whereas ventricular hemorrhage may induce ventricular wall fibrosis and malfunction, both of which are closely related to impaired CSF absorption. Low Glasgow Coma Score (GCS ≤ 8) and high Injury Severity Score (ISS ≥25) on admission are recognized as independent risk factors for PTH, indicating that patients with severe craniocerebral injuries should be closely monitored for the potential development of secondary hydrocephalus (1, 53–55).

#### 4.2 Surgery-related factors

Secondly, elements associated with surgical intervention significantly influence the incidence of PTH. Decompressive craniectomy (DC) is a prevalent surgical intervention for severe craniocerebral injuries, and DC is a strong predictor of PTH. Numerous studies indicate that the incidence of PTH after DC may reach 11.9%−45%, much higher than that observed in non-surgical patients (1, 4, 6, 22, 56). Meta-analysis indicates that DC results in an imbalance of cranial cavity volume and disruption of CSF dynamics, whereas subdural effusion and brain tissue collapse exacerbate CSF circulation problems (57). In addition, surgical complications such as brain infections have been associated with an increased incidence of PTH, potentially due to the release of inflammatory mediators (58, 59). A research indicated that early cranioplasty (within 2 months after DC) decreased the likelihood of PTH (60).

#### 4.3 Other factors

Imaging symptoms possess significant early warning potential for PTH. Subdural effusion constitutes an independent risk factor for PTH (59). Midline shift > 5 mm and ventricular enlargement indicate heightened CSF outflow resistance, necessitating caution for potential delayed PTH (6). Intraventricular hemorrhage may promote the development of PTH by obstructing the CSF circulatory channel or hindering CSF absorption due to blood breakdown products (61). Nonetheless, it has been proposed that this is indeed statistically non-significant (62). Traumatic subarachnoid hemorrhage is significantly correlated with the formation of PTH, likely because CSF dynamics are influenced by hydrostatic pressure and osmotic forces, capillaries play a major role in CSF production and absorption owing to their large surface area, the rupture of subarachnoid capillaries results in compromised CSF absorption, blood accumulation in the basal pool obstructs CSF flow, and fibrosis in the subarachnoid space may also contribute to PTH formation (62).

Age is a bidirectional risk factor for PTH, with children ( ≤ 5 years) prone to PTH due to immaturity of the CSF regulatory system and older patients (>80 years) also at increased risk due to cerebral atrophy and decreased compensatory capacity (1, 63–65). Preexisting conditions, including diabetes mellitus (OR = 1.5) indirectly contribute to the onset of PTH by aggravating cerebral edema and inflammatory responses through microangiopathy (4). Beyond chronological age alone, biological aging factors such as pre-existing cerebral small-vessel disease, prior subclinical TBI, reduced AQP4 polarization, and accumulation of perivascular protein aggregates (amyloid-β and phosphorylated tau) plausibly lower the threshold at which post-injury CSF disturbance progresses to overt PTH, and may also help to explain the cognitive decline frequently observed in older PTH survivors. These aging and comorbidity factors should therefore be incorporated into individual risk stratification rather than considered as fixed demographic variables.

### 5 New advances in diagnostic techniques

In recent years, diagnostic technology for PTH has advanced significantly in multimodal imaging evaluation, biomarker identification, and artificial intelligence-enhanced analysis. Diagnostic imaging is primarily focused on CT and MRI; nevertheless, the use of parametric analysis has notably improved early detection. The European expert agreement is that patients with cranial abnormalities and suspected PTH require a thorough clinical neurological evaluation accompanied by serial imaging follow-up to detect early indications of increasing ventricular enlargement (66). An Evans index > 0.3 has been shown to be a sensitive imaging marker for PTH, particularly in individuals with cranial abnormalities (67). The CT ventricular volume automated measurement system, integrated with artificial intelligence algorithms, efficiently computes the Evans index and ventricular dilation trends, thereby enhancing diagnostic efficacy and objectivity, particularly for rapid evaluations in the emergency contexts (68). The integration of artificial intelligence technologies with essential neurological and neurological biomarkers can enhance diagnostic efficacy. imaging biomarkers can assist clinicians in creating effective and precise diagnostic instruments (68). The investigation of biomarkers has offered a novel approach for diagnosing PTH. Zhang et al. (69) found that plasma S100B levels were significantly correlated with PTH, suggesting that S100B may be used as a secondary diagnostic marker for PTH, but unfortunately, S100B cannot be used as an early screening indicator. Temiz et al. discerned potential alterations in carnitine metabolism by contrasting the carnitine profiles of children with hydrocephalus against those of healthy children, demonstrating that the findings suggest modifications in carnitine metabolism may occur in hydrocephalus. The findings regarding potential alterations in carnitine indicated that 23 carnitine levels were markedly diminished in the hydrocephalus cohort. It was ultimately established that C12, with a specificity of 100% and a sensitivity of 80%, may be used as a secondary diagnostic marker for hydrocephalus (70). Despite the progress made in biomarker research and AI-based analyses, large-scale prospective studies are still needed to confirm the reliability and applicability of these results. White matter injury, evaluated by diffusion tensor imaging (DTI) metrics such as fractional anisotropy and mean diffusivity in the periventricular white matter and corpus callosum, has been reported to track ventricular distension and to correlate with post-shunt cognitive recovery, suggesting an additional imaging dimension for early diagnosis and prognosis. The combination of conventional morphometric indices, DTI-based white-matter signatures, fluid biomarkers (S100B, neurofilament light chain), and machine-learning algorithms is moving toward a multimodal, individualized diagnostic framework for PTH.

### 6 Treatment

#### 6.1 Surgery

The surgical management of PTH involves cerebrospinal fluid shunts, including ventriculoperitoneal shunts (VPS), ventriculoventricular shunts (VAS), lumbar-peritoneal shunts (LPS), and endoscopic third ventriculostomy (ETV), with VPS being the clinically preferred option (14). Kowalski et al. (71) demonstrated that early VPS (median time 10 days post-injury) significantly reduced the length of posttraumatic amnesia and enhanced the Functional Independence Score. The risk of postoperative complications following surgical therapy is around 20%−22%, and mainly involves hemorrhage, infection, and shunt obstruction (18, 72). The 2024 European Expert Consensus advises that for patients with combined cranial defects, cranioplasty should be prioritized, followed by an assessment of whether to implement VPS based on alterations in ventricular dynamics, to reduce the risk of postoperative subdural effusion and brain herniation (66). In recent years, ETV has been increasingly investigated as an alternative, particularly for patients with aqueductal stenosis or recurrent shunt failures. However, a comparative study by Sharma et al. (73) revealed a much lower clinical improvement rate for ETV (37%) compared to VPS (73%), along with a higher rate of postoperative re-intervention (37 vs. 13%), indicating that VPS remains the more dependable option. The preferred therapy for PTH, either ETV or VPS may require additional validation through subsequent trials. In difficult circumstances when ETV or VPS is unfeasible due to the condition, a customized shunt may offer a tailored solution. Lee et al. documented a case in which a patient, unable to receive conventional VPS due to heart failure, was effectively drained of of CSF via ventriculo-vesical shunt (VVS). Three months post-operation, a reduction in ventricular size was observed without urinary complications, indicating that VVS may serve as a viable alternative to VPS in scenarios involving limited abdominal cavity conditions (74). In summary, advancements in the surgical treatment of PTH primarily involve the refinement of established techniques (e.g., VPS, ETV) and the handling of complications, while the introduction of novel procedures like VVS may provide a significant adjunct to PTH in instances where VPS and ETV are unfeasible.

#### 6.2 Non-surgical treatment

Non-surgical intervention mostly targets mild or early PTH. In the 1870s, researchers identified that isosorbide could temporarily regulate intracranial pressure, making it a prevalent medication for treating hydrocephalus during that period; however, its adverse effect of hypertonic dehydration eventually curtailed its use (75), Steroids, osmotic agents (e.g., Manitol), diuretics (e.g., Fosamax), and acetazolamide can suppress CSF secretion, thereby serving as primary agents for reducing intracranial pressure. However, due to the risk of rebound cerebral hypertension, Mannitol should not be administered for more than 72 h, acetazolamide and fosfomycin for no more than 4 weeks, and dexamethasone for a maximum of 6 weeks (76). Recently, Zhu et al. (10) documented a 16% reduction in Evans' index and notable enhancement in motor and cognitive function following oral administration of atorvastatin (20 mg/day) in a patient with PTH, indicating that anti-inflammatory and neuroprotective effects may impede the progression of hydrocephalus, however, this type of pharmacological intervention requires validation through large-scale randomized controlled trials. In conclusion, thus far, no medication has demonstrated a significant enhancement of hydrocephalus symptoms or a reversal of illness development. Pharmacological management of PTH is still at an early stage and faces two main challenges: limited individualization and a relatively high complication rate. Further research is essential to create compounds with more targeted and specific characteristics that could progressively supplant surgical interventions (77).

#### 6.3 Mechanism-targeted therapeutic strategies

In parallel with surgical refinement, several mechanism-based strategies are being explored as adjuncts to shunting. Anti-inflammatory interventions targeting the TLR4/NF-κB axis, the NLRP3 inflammasome, and IL-1β/IL-18 signaling aim to attenuate choroid plexus hypersecretion and ependymal injury; iron chelators (deferoxamine and minocycline-related agents) are being investigated to reduce haem-iron toxicity after subarachnoid or intraventricular hemorrhage; AQP4 modulators and glymphatic-restoration approaches (sleep hygiene, head-of-bed positioning, candidate small molecules) seek to re-establish perivascular clearance; SGK1 inhibitors and Na^+^/K+/2Cl^−^ cotransporter blockade target the secretory pathway directly; and ciliary or genetic stratification (RGS22, DNAH5, ULK4, and CEP290) opens a path toward precision risk classification. None of these approaches is yet ready for routine clinical use, but together they outline a translational research agenda that connects the pathophysiological mechanisms discussed above to future targeted interventions (39, 77).

### 7 Prevention and management

The timeline for the onset of PTH is highly variable; thus, its prevention and management must occur throughout the entire trajectory of TBI. Early monitoring and interdisciplinary collaboration are therefore needed across a window from one week after injury up to 31.5 months, with the first 50 days being particularly important (65). Other studies suggest that heightened awareness is essential for PTH development during the first 102 days following hospital discharge (6). The research conducted by Akira et al. indicates that individuals with severe TBI ought to undergo routine imaging assessments (e.g., CT or MRI), alongside dynamic monitoring of ventricular enlargement (Evans index) and fluctuations in intracranial pressure, to facilitate the early detection of PTH risk. The neurorehabilitation team should concentrate on high-risk patients exhibiting combined skull abnormalities, intraventricular hemorrhage, or desmoidal decompression, since these conditions may result in a 7–%−10% incidence of PTH within the cohort. Enhancing perioperative care (e.g., infection control, reduction of CSF trait abnormalities) may diminish the risk of PTH (78).

Upon confirmation of PTH diagnosis, management techniques must be tailored and emphasize functional rehabilitation (79). The European Consensus (2024) advises that in patients with cranial abnormalities accompanied by PTH, priority should be given to performing cranioplasty to restore the physiological intracranial pressure gradient prior to determining the necessity of shunt surgery based on a kinetic evaluation of the CSF (66). Postoperative neurorehabilitation (e.g., cognitive behavioral therapy, gait training) needs to be combined to improve neurological deficits, and the progress of recovery is regularly assessed by the Glasgow Prognostic Scale (GOS). Multidisciplinary collaboration (neurosurgery, rehabilitation, endocrinology) is useful in addressing PTH-related problems, including neuroendocrine abnormalities and autonomic hyperfunction (79).

Effective prevention of PTH requires a multi-pronged approach, incorporating primary prevention of TBI, risk management, and tailored measures to reduce both the incidence and severity of PTH (80). Nonetheless, problems persist, and it is anticipated that advancements in understanding the causes of PTH in forthcoming research will lead to significant progress in ultra-early detection, non-invasive therapies, and the prevention of clinical PTH (77). Beyond conventional perioperative care and clinical surveillance, the next generation of preventive strategies should explicitly target the secondary-injury cascade. Candidate approaches include early anti-inflammatory intervention against the TLR4/NF-κB and NLRP3 axes, iron chelation after subarachnoid or intraventricular hemorrhage, restoration of glymphatic flow and AQP4 polarization, neuroprotective therapies addressing oxidative stress and white-matter injury, and biomarker-guided early intervention using fluid markers (S100B, neurofilament light chain) together with AI-assisted imaging to identify high-risk patients before overt ventriculomegaly develops. Combining mechanistic understanding with such precision-prevention tools should help shift PTH management from late rescue surgery toward earlier, individualized disease modification.

#### 7.1 Highlights and future perspectives

Looking ahead, three areas appear most likely to reshape clinical management of PTH. (i) Multimodal diagnostics combining DTI-based white-matter signatures, AI-driven morphometric analysis, and fluid biomarkers (S100B, neurofilament light chain, inflammasome-related cytokines) are expected to enable risk stratification within the first weeks after TBI. (ii) Genetic and molecular profiling of ciliary genes (DNAH5, RGS22, ULK4, and CEP290), inflammatory signaling components, and astrocytic AQP4 status may move PTH care toward personalized medicine and identify candidates for targeted prophylaxis. (iii) Mechanism-based disease modification, including selective anti-inflammatory therapy, iron chelation, glymphatic restoration during sleep, and small-molecule modulators of CSF secretion (SGK1 inhibitors, NKCC1 antagonists), offers a route to complement, and potentially delay, shunt surgery. Adequately powered prospective and multicentre studies that integrate these dimensions will be essential to translate emerging biology into durable clinical benefit.
